# Significance of Follicle-Stimulating Hormone Receptor Gene Single-Nucleotide Polymorphism rs6165/rs6166 Analysis for Infertility-Associated Ovarian Disease Susceptibility Prediction and Optimized Individualized Ovulation Induction/Ovarian Stimulation

**DOI:** 10.3390/diagnostics16020221

**Published:** 2026-01-10

**Authors:** Kotaro Kitaya, Atsumi Hamazaki, Naoko Kobayashi, Takako Mihara, Masaya Mihara

**Affiliations:** 1 Infertility Center, Iryouhoujin Kouseikai Mihara Hospital, 6-8 Kamikatsura Miyanogo-cho, Nishikyo-ku, Kyoto 615-8227, Japan; 2 Iryouhoujin Kouseikai Katsura-ekimae Mihara Clinic, 103 Katsura OS Plaza Building, 133 Katsura Minamitatsumi-cho, Nishikyo-ku, Kyoto 615-8074, Japan

**Keywords:** ART, follicle-stimulating hormone receptor, polymorphism, IVF/ICSI, infertility, ovarian stimulation

## Abstract

Follicle-stimulating hormone receptor (FSHR) is expressed on the plasma membrane of granulosa cells in the ovarian follicles. FSHR is involved in the development and maturation of Graafian follicles, along with granulosa proliferation and estrogen synthesis. There are two well-characterized non-synonymous single-nucleotide gene polymorphisms in the exon 10 of the human FSHR gene, namely rs6165 (c.919G>A, Ala307Thr) and rs6166 (c.2039A>G, Ser680Asn). Recent research clarifies the association of rs6165/rs6166 with susceptibility to infertility-associated ovarian diseases, ranging from polycystic ovarian syndrome, premature ovarian insufficiency, endometriosis, to ovarian cancer, along with response/resistance to ovulation induction/ovarian stimulation with clomiphene citrate, letrozole, metformin, FSH preparations, and adjunctive growth hormone in infertility treatment. This narrative review aims to update the knowledge on the relationship among rs6165/rs6166, infertility etiology, and differential responses to oral ovulation induction agents, FSH preparations, and adjunctive growth hormone. The re6165/rs6166 genotype-guided choice of individualized ovulation stimulation preparations has great potential to reduce unexpected poor or high ovarian responses in ovulation induction and ovarian stimulation and improve clinical outcomes in reproductive medicine. Current evidence is insufficient, and further studies are warranted to ascertain its potential for clinical implementation.

## 1. Introduction

Follicle-stimulating hormone receptor (FSHR) is a seven-transmembrane type G-protein-coupled receptor, of which cDNA was first cloned from rat testicular Sertoli cells [1]. FSHR protein consists of 695 amino acids and has a molecular mass of approximately 76 kDa. In humans, FSHR is physiologically expressed on the plasma membrane of granulosa cells in the ovary, Sertoli cells in the testis, and epithelial and stromal cells in the endometrium. FSHR binds FSH, a glycoprotein polypeptide hormone that is synthesized and secreted by the gonadotropic cells in the anterior pituitary gland. The interaction of FSHR with FSH stimulates the development, growth, pubertal maturation, and reproductive processes of the body [2,3,4,5].

Male mice lacking the *fshr* gene are fertile, despite partial spermatogenic failure accompanied by reduced testicular size and seminiferous tubule density. Conversely, the female mice lacking the *fshr* gene are infertile with hypogenesis of the uterus and ovaries. The ovaries in female *fshr* gene knockout mice contain primordial, primary, and secondary follicles, but not large Graafian follicles, indicating a crucial role of *fshr* signaling in late follicular development and maturation [6,7]. The human FSHR gene is located on chromosome 2p21 and comprises approximately 2080 nucleotides [8]. The human *fshr* gene is approximately 192 kb in size and consists of ten exons and nine introns. The exons 1–9 encode the N-terminal part of the extracellular domain of the receptor, whereas exon 10 encodes the C-terminal part (hinge region) of the extracellular domain, transmembrane domain, and intracellular domain [9]. The activity of the *fshr* gene is driven by a core promoter spanning 225 base pairs, which represents a TATA-less promoter without evident regulatory elements except for an E-box and a more recently identified initiator element [10].

The spliced variants of the human *fshr* gene have been reported. Some of these variants potentially cause female infertility with polycystic ovarian syndrome, hypergonadotropic ovarian dysgenesis, primary ovarian failure, and diminished ovarian reserve [11]. Additionally, there are two well-characterized non-synonymous single-nucleotide polymorphisms in the *fshr* gene, concerning allele frequencies and ethnic distribution. One is rs6165 (c.919G>A) polymorphism in the hinge region of the extracellular domain, which has an allele G at position 919, leading to codon 307 substitution from alanine (GCT) to threonine (ACT). The rs6165 G-to-A substitution results in the alteration from a polar to a nonpolar, hydrophobic amino acid and the removal of a potential O-linked glycosylation site, which may affect FSH binding affinity and ovarian response [12,13]. The other is rs6166 (c.2039A>G) polymorphism in the intracellular domain, which has an allele G at position 2039, resulting in codon 680 substitution from serine (AGT) to asparagine (AAT). The rs6166 A-to-G substitution potentially alters the phosphorylation site in the intracellular domain of the receptor, which may also influence FSH binding affinity and ovarian response [12,14,15]. rs6165 and rs6166 are linked to each other during recombination [12]. The frequency distribution studies demonstrated that Thr307-Asn680 (~60%) and Ala307-Ser680 (~40%) are very common in men and women in various populations, whereas Ala307-Asn680 (~5%) and Thr307-Ser680 (~5%) are found only sporadically [16,17].

In this narrative review, we aimed to address the current literature surrounding rs6165/rs6166, infertility-associated ovarian diseases, and ovarian response/resistance to ovulation induction agents and ovarian stimulation preparations.

## 2. Methods

The following databases were searched for articles: PubMed (the National Institutes of Health, MD, USA), Google Scholar (Google Inc., Mountain View, CA, USA), and Embase/ ScienceDirect (Elsevier, Amsterdam, The Netherlands). Each database was searched using the following terms: “FSHR”, “FSHR polymorphism”, “rs6165”, “rs6166”, “Ala307Thr”, “Asn680Ser”, “polycystic ovarian syndrome”, “premature ovarian insufficiency”, “endometriosis”, “ovarian cancer”, “ovarian stimulation”, “ovulation induction”, “clomiphene citrate”, “letrozole”, “metformin”, “recombinant FSH”, “urinary FSH”, “growth hormone” and “infertility” from their inception to October, 2025. Only English literature was included.

## 3. rs6165/6166 and Infertility-Associated Ovarian Disorders

### 3.1. rs6165/6166 and Polycystic Ovarian Syndrome

Polycystic ovary syndrome (PCOS) is an endocrinological disorder characterized by anovulation/oligo ovulation, hyperandrogenism [elevated serum testosterone and luteinizing hormone (LH) levels and/or clinical features such as hirsutism and acne], and polycystic ovarian morphology on ultrasound [18]. PCOS is also associated with metabolic disturbance, including obesity, insulin resistance, and hyperinsulinemia; therefore, it is recognized as a risk factor for type 2 diabetes and cardiovascular disease in later life. PCOS is estimated to affect ~20% of women of reproductive age [19].

Accumulating studies investigated the relationship between rs6165/rs6166 and susceptibility to PCOS, although the results are inconsistent [20,21,22,23,24,25]. While the majority of Chinese and European studies, along with one Thai study, do not substantiate the relationship between PCOS and rs6165/rs6166, some Korean and Caucasian studies disclosed that the rs6166 AA (Asn680/Asn680) and AG (Asn680/Ser680) genotypes are associated with a lower risk for PCOS. Moreover, some Chinese, Korean, Japanese, Pakistani, and Dutch studies indicate that the rs6165 AA (Ala307/Ala307) and AG (Ala307/Thr307) as well as the rs6166 GG (Ser680/Ser680) and AG (Asn680/Ser680) genotypes are associated with an increased risk of developing PCOS. The most recent meta-analysis included the highest number of studies ever for fixed-effect models. Using dominant model [GG (Thr307/Thr307) + AG (Ala307/Thr307) vs. AA (Ala307/Ala307)]: OR = 1.04, 95% CI: 0.93–1.16, *p* = 0.49; recessive model [GG (Thr307/Thr307) vs. AG (Ala307/Thr307) + AA (Ala307/Ala307)]: OR-1.19 95% CI: 1.03–1.3, *p* = 0.02; additive model [GG (Thr307/Thr307) vs. AA (Ala307/Ala307)]: OR-1.2 95% CI: 1.02–1.42, *p* = 0.03; and allele model [G (Thr307) vs. A (Ala307)]: OR = 1.07, 95% CI: 0.99–1.18, *p* = 0.08, this study demonstrated that rs6165 does not exhibit an association with PCOS in analysis of 14 studies with 2650 cases and 3226 controls (Table 1) [26].

Meanwhile, the rs6166 GG (Ser680/Ser680) and AG (Asn680/Ser680) genotypes are associated with a low to moderate risk for PCOS in Caucasian populations in the allele model (OR = 1.17, 95% CI: 1.04–1.32, *p* = 0.01), but not in Asian populations [26]. A comparative study from the Netherlands investigated the rs6166 in 1771 women of five different ethnic origins, including 1431 Caucasian, 67 Asian, 33 Hindustani, 65 Creole, and Mediterranean women who visited the reproductive medicine unit [27]. The frequency of the rs6166 GG (Ser680/Ser680) and AG (Asn680/Ser680) genotypes in the Caucasian (21.5% and 48.9%, respectively) and Mediterranean (22.3% and 51.4%) populations was higher than in Asian (10.5% and 38.8%, Japanese and Chinese) populations (*p* = 0.010) [27]. Additionally, under the Rotterdam criteria [18], the prevalence of PCOS in Caucasian populations (18%) is generally reported to be higher than in Asian populations (ranges from 5% to 19%, depending on ethnicity) [28,29], despite marked variances among the studies. There may be some associations between the rs6166 GG (Ser680/Ser680) and AG (Asn680/Ser680) genotypes and susceptibility to PCOS.

Intriguingly, the rs6166 AA (Asn680/Asn680) and AG (Asn680/Ser680) genotypes are protective against PCOS in the Indian population (19 studies with 3671 cases and 6579 controls) in the recessive model [GG (Ser680/Ser680) vs. AG (Asn680/Ser680) + AA (Asn680/Asn680)]: OR = 0.7, 95% CI: 0.54–0.9, *p* = 0.006, additive model [GG (Ser680/Ser680) vs. AA (Asn680/Asn680)]: OR = 0.65, 95% CI: 0.48–0.89, *p* = 0.006, and allele model [G (Ser680) vs. A (Asn680)]: OR = 0.82, 95% CI: 0.7–0.95, *p* = 0.01, but not in other Asian populations [26]. The prevalence of PCOS due to the Rotterdam Criteria in Indian women (19.6%, 95% CI 12.7–29.2%) was reported to be highest compared with other Asian women (5–19%), with phenotype C (hyperandrogenism and polycystic ovarian morphology) being the most prevalent (40.8%) [29]. Meanwhile, another study claimed that the rs6166 AA (Asn680/Asn680)] genotype is more common in Indian PCOS women with phenotype D (non-hyperandrogenism and higher LH/FSH ratio) [30]. The relationship between rs6166 and PCOS in the Indian population seems to be complicated. Further studies are required for clarification.

Thus, rs6166 is likely to have a modest impact on PCOS in a specific ethnic sub-cohort, but the results cannot be generalized due to the high heterogeneity of rs6166.

**Table 1:** Summary of meta-analysis on the association between FSHR polymorphisms rs6165/6166 and onset of infertility-associated ovarian disorders.

	rs6165	rs6166
PCOS	No apparent association (meta-analysis) [26]	AA (Asn680/Asn680) and AG (Asn680/Ser680); associated with reduced risk of onset in the Indian population but not in other Asian populations (meta-analysis) [26] GG (Ser680/Ser680) and AG (Asn680/Ser680); associated with low to moderate risk of onset for PCOS in Caucasian populations, but not in Asian populations (meta-analysis) [26]
POI	No apparent association (meta-analysis) [31,32]	GG (Ser680/Ser680) and AG (Asn680/Ser680); associated with increased risk of onset in Asian populations (meta-analysis) [31,32]
Endometriosis	No apparent association (case–control studies) [33,34,35,36,37]	No apparent association (meta-analysis) [38]
Ovarian cancer	AA (Ala307/Ala307); associated with increased risk of onset of serous and mucinous ovarian carcinoma (case–control studies) [39,40]	GG (Ser680/Ser680) and AG (Asn680/Ser680); associated with increased risk of onset in Asian populations (meta-analysis) [41]

### 3.2. rs6165/rs6166 and Premature Ovarian Insufficiency

Premature ovarian insufficiency (POI) is defined as an apparent loss of ovarian function before the age of 40. POI is characterized by amenorrhea or irregular menstrual cycles with elevated gonadotropins (FSH > 25 IU/L) and low estradiol levels. According to epidemiological studies, the prevalence of POI varies from approximately 1% in older studies to 3.5% in recent publications. Recent studies highlight the involvement of genetic factors in POI [42,43]. The other risk factors of POI include iatrogenic gynecological surgical practice, lifestyle factors such as cigarette smoking and binge drinking, and treatment regimens for malignant and chronic diseases [44].

The results of the two meta-analyses (one analysis including 14 studies with 590 cases and 1170 controls, and another analysis including 9 studies with 558 patients and 1119 controls) agree that POI is not associated with rs6165 (Table 1) [31,32]. Studies also indicate that POI is unassociated with rs6166 in the overall analysis (13 studies with 640 cases and 1333 controls), either. A subgroup analysis, however, demonstrated that the rs6166 GG (Ser680/Ser680) and AG (Asn680/Ser680) genotypes were associated with an increased risk of POI only in Asian populations, as shown in an additive comparison in both the fixed-effect model and random-effect model (Table 1) [31]. The rs6166 GG genotype (Ser680/Ser680) has been associated with higher serum FSH, lower serum estradiol concentrations, and longer follicular phase length compared with the AA genotype (Asn680/Asn680) [45]. rs6166 may potentially affect ovarian reserve in some ethnic groups.

The association between the ethnic differences in rs6166 and susceptibility to POI is an intriguing subject. The frequency of rs6166 GG (Ser680/Ser680) was found to be much lower in Asian (10.5%, Japanese and Chinese) infertile women than in the Caucasian (21.5%) and Mediterranean (22.3%) counterparts (*p* = 0.010), despite no statistical difference in serum FSH concentration. Meanwhile, a meta-analysis disclosed that the prevalence of POI is lower in the Asian population (3.3%, 95% CI: 2.1–4.5%, I^2^ = 89.9%, *p* < 0.01) compared with North American (11.3%, 95% CI: 9.5–13.1%, I^2^ = 0.0%, *p* < 0.37) and South American (5.4%, 95% CI: 4.0–6.8%, I^2^ = 0.0%) populations, although it was at a similar level to Europeans (2.3%, 95% CI: 1.9–2.8%, I^2^ = 94.3%, *p* < 0.01). While the causality between rs6166 GG (Ser680/Ser680) and low prevalence in POI remains undetermined, the relationship between rs6166 and susceptibility to POI is worth exploring.

### 3.3. rs6165/6166 and Endometriosis

Endometriosis is an estrogen-dependent chronic, inflammatory disease characterized by the ectopic growth of the endometrioid tissues outside the uterine cavity. While the common sites of endometriosis are the ovaries, fallopian tubes, and peritoneum, ectopic endometrioid tissues occasionally can develop further into extrapelvic organs. Endometriosis affects up to 10% of women of reproductive age worldwide. 90% of women with endometriosis report pelvic pain, including dysmenorrhea, non-menstrual pelvic pain, dyspareunia, dyschezia, and dysuria. Risk factors for endometriosis include younger age at menarche, shorter menstrual cycle length, lower body mass index, nulliparity, and congenital obstructive müllerian anomalies. While laparoscopy is essential for definitive diagnosis of endometriosis, a suspected clinical diagnosis can be made based on symptoms, physical examinations, and medical imaging with transvaginal ultrasound and/or pelvic magnetic resonance. A quarter of women with endometriosis suffer from infertility. Early intervention may be considered for women with endometriosis who desire pregnancy. The European Society of Human Reproduction and Embryology guideline 2022 recommends intrauterine insemination with controlled ovarian stimulation for women with the revised American Society for Reproductive Medicine stage I/II (minimal/mild) endometriosis and good ovarian reserve and without significant male factor infertility or tubal occlusion/stenosis, as a first-line treatment to improve live birth rates. For patients with stage III/IV (moderate/severe) endometriosis or those complicated with compromised tubal function and diminished ovarian reserve, assisted reproductive technology should be considered [46].

Studies failed to establish a clear association between rs6165 and the onset of endometriosis. In 2011 study of Taiwanese Chinese population, the rs6165 allele frequency was at a similar level between 300 women with endometriosis and 337 women without endometriosis [46.7% vs. 46.3% in the AA (Ala307/Ala307), 40.7% vs. 40.0% in the AG (Ala307/Thr307), and 12.7% vs. 13.7% in the GG (Thr307/Thr307), *p* > 0.07] genotypes [33,34]. Furthermore, in a Brazilian study, there were no differences in the rs6165 allele frequency [28.4% vs. 24.1% in the AA (Ala307/Ala307), 47.5% vs. 51.4% in AG (Ala307/Thr307), and 24.5% vs. 24.1% in GG (Thr307/Thr307) genotypes, *p* = 0.358] between 352 women with endometriosis and 510 women without endometriosis [35]. These results are consistent with other studies of smaller sample sizes targeting different ethnicities. In a Romania Caucasian study, researchers did not find any statistical differences in the rs6165 allele frequency between 44 women with endometriosis and diminished ovarian reserve and those without endometriosis [38.6% vs. 23.5% in the AA (Ala307/Ala307), 45.5% vs. 67.6% in the AG (Ala307/Thr307), and 15.9% vs. 8.8% in the GG (Thr307/Thr307) genotypes, *p* = 0.295] [36]. In a Turkish population, the rs6165 allele frequency was also similar between 100 women with endometriosis and 100 women without endometriosis [22.0% vs. 14.0% in the AA (Ala307/Ala307), 54.0% vs. 65.0% in the AG (Ala307/Thr307), and 24.0% vs. 21.0% in the GG (Thr307/Thr307) genotypes, *p* > 0.364] [37]. However, sub-cohort analysis by this Turkish group disclosed that the rs6165 AA (Ala307/Ala307) genotype was less likely to develop stage III–IV endometriosis than the AG (Ala307/Thr307) and GG (Thr307/Thr307) genotypes (15,8% vs. 57.9% vs. 26.3%, OR 0.177, 95% CI 0.055–0.568, *p* = 0.004) [37]. The rs6165 AG (Ala307/Thr307) and GG (Thr307/Thr307) genotypes are therefore unlikely to relate to the onset of endometriosis, but may be associated with disease progression.

There are some inconsistencies in the literature on the relationship between rs6166 and endometriosis. In a Taiwanese Chinese study, the rs6166 AA (Asn680/Asn680) genotype was associated with an increased risk for endometriosis (49.3% in the endometriosis group vs. 37.4% in the non-endometriosis group), whereas the rs6166 GG (Ser680/Ser680) (40.3% vs. 51.0%, OR 0.75, 95% CI 0.59–0.95, *p* = 0.02) and AG (Asn680/Ser680) (11.3% vs. 10.3%, OR 0.60, 95% CI 0.42–0.84, *p* = 0.002) genotypes were associated with a decreased risk, especially in women with endometriosis aged younger than 37 years old [33]. By contrast, in a Brazilian prospective study, there were no differences in the rs6166 allele frequency between 67 infertile women with endometriosis and 65 healthy fertile women [~35% vs. ~30% in the AA (Asn680/Asn680), ~50% vs. ~55% in the AG (Asn680/Ser680), and ~15% vs. ~15% in the GG (Ser680/Ser680) genotypes, *p* = 0.78] [47]. In addition, in another Brazilian study, there were no differences in the rs6166 allele frequency [35.3% vs. 34.1% in the AA (Asn680/Asn680), 49.8% vs. 46.3% in the AG (Asn680/Ser680), and 14.9% vs. 19.6% in the GG (Ser680/Ser680) genotypes, *p* = 0.129] between 352 women with endometriosis and 510 women without endometriosis [35]. A meta-analysis of the three studies where the absence of endometriosis in the control group was confirmed during laparoscopy or laparotomy identified no associations between rs6166 and endometriosis in a fixed effect model without publication bias (OR 1.18; 95% CI 0.99–1.41) (Table 1) [38]. However, sub-cohort analysis by the Turkish group found that the rs6166 GG (Ser680/Ser680) genotype was less likely to develop stage III–IV endometriosis compared with the rs6166 AA (Asn680/Asn680) genotype (OR 0.177, 95% CI 0.055–0.568, *p* = 0.00) [37]. Meanwhile, the Brazilian group demonstrated that the rs6166 GG (Ser680/Ser680) genotype was less frequent in fertile women with endometriosis than in infertile counterparts (17.9% vs. 23.2%, OR 1.81, 95% CI 1.02–3.22, *p* = 0.041), despite there were no differences in the frequency of the other two genotypes [46.4% vs. 46.3% in the AG (Asn680/Ser680) and 34.1% vs. 30.4% in the GG (Ser680/Ser680) genotypes, *p* = 0.737]. Interestingly, these findings are independent of the stage of endometriosis [35]. Thus, the association of rs6166 with disease onset, disease progression, and infertility status in endometriosis does not seem to be simple.

### 3.4. rs6165/6166 and Ovarian Cancer

Ovarian cancer is the eighth-most common female cancer worldwide. Approximately 90% of ovarian cancers are epithelial malignancies, of which 75% are high-grade serous epithelial carcinoma. Risk factors for ovarian cancer include aging (63 years, median age at diagnosis), family history of breast or ovarian cancer, endometriosis (particularly ovarian endometrioma), nulliparity, and hereditary factors predominantly linked to BRCA1/2 gene variants. 80% of patients have advanced-stage (stage III–IV) lesions. Although the methods for fertility preservation are limited, successful ex vivo follicular puncture and aspiration from intact, unruptured oophorectomized specimens are reported [48].

A Chinese–Han study from Hong Kong found that women with the rs6165 AA (Ala307/Ala307) and rs6166 GG (Ser680/Ser680) genotypes are a higher risk of developing serous ovarian carcinoma (OR = 2.60, 95% CI = 1.56–4.34, *p* < 0.0005) and mucinous ovarian carcinoma (OR = 2.89, 95% CI = 1.73–4.84, *p* < 0.0005) (not of endometrioid and clear cell ovarian carcinoma) compared with other two respective genotypes even after adjustment for age. The rs6165 AA (Ala307/Ala307) and rs6166 GG (Ser680/Ser680) genotypes were in modest linkage disequilibrium with higher ovarian cancer risk and the major haplotype Ala307-Ser680 was also associated with ovarian cancer risk (*p* = 0.033, OR = 1.39, 95% CI = 1.03–1.88), particularly with the serous and mucinous ovarian carcinomas (*p* = 0.001, OR = 1.82, 95% CI = 1.27–2.60) [39]. Moreover, a Polish Caucasian study also found that women with the rs6165 AA (Ala307/Ala307) (18.69%) (1.84-fold) had a higher risk for ovarian carcinoma compared with other genotypes [40]. Additionally, the rs6165 AA (Ala307/Ala307) genotype showed a tendency to diminish odds of complete remission (OR 0.24, 95% CI 0.06–1.06, *p* = 0.059).

The association between rs6166 and ovarian cancer risk remains controversial. Granulosa cell tumors are rare sex cord-stromal tumors. Granulosa cell tumors arise from granulosa cells surrounding oocytes and exhibit many features of normal granulosa cells by secreting estrogens in response to FSH and inhibin. Serum FSH concentration is low in patients with inhibin-secreting granulosa cell tumors, suggesting an FSH-independent growth of tumors. Thus, activating mutations of the *fshr* gene may be associated with carcinogenesis. A Caucasian study from Australia that enrolled 22 women with granulosa cell tumors, however, failed to demonstrate a clear association of rs6166 with its onset [49]. By contrast, a Polish Caucasian study also found that women with the rs6166 GG (Ser680/Ser680) genotypes (18.87%) (1.82-fold) had a higher risk for ovarian carcinoma compared with other genotypes. The subgroup analysis disclosed an increased risk of serous ovarian carcinoma for the rs6166 GG (Ser680/Ser680) genotype (OR 1.9, 95% CI (1.02; 3.59), *p* = 0.042). Furthermore, the rs6166 GG (Ser680/Ser680) genotype showed a tendency for nearly twice the risk of recurrence (*p* = 0.062) and death (*p* = 0.06) compared with the whole group [40]. Finally, a meta-analysis included the three above-mentioned case–control studies and one additional case–control study (written in Chinese, a total of 474 ovarian cancer patients and 659 controls) [41,50]. This study demonstrated that the rs6166 GG genotype (Ser680/Ser680) is associated with a higher risk of ovarian cancer compared with rs6166 AG (Asn680/Ser680) and AA (Asn680/Asn680) genotypes (Ser680 vs. Asn680: OR = 1.295, 95% CI 1.057–1.498, *p* = 0.01; Ser680/Ser680 + Asn680/Ser680 vs. Asn680/Asn680: OR = 1.611, 95% CI 1.027–2.528, *p* = 0.038). Interestingly, subgroup analyses revealed an association between the rs6166 GG genotype (Ser680/Ser680) and ovarian cancer in Asians (Ser680 vs. Asn680: OR = 1.386, 95% CI 1.066–1.802, *p* = 0.015; Ser680/Ser680 + Asn680/Ser680 vs. Asn680/Asn680: OR = 1.893, 95% CI 1.329–2.689, *p* = 0.000) but not in Caucasians (Table 1). However, the authors concluded that due to the limited number of patients, further studies are required to validate the results [41].

## 4. rs6165/6166 and Ovarian Response/Resistance to Ovulation Induction Agents and Ovarian Stimulation Preparations

### 4.1. rs6165/6166 and Ovarian Response/Resistance to Clomiphene Citrate

Clomiphene citrate (CC) is an oral selective estrogen receptor modulator/nonsteroidal triphenylethylene derivative that was developed as MRL/41 by William S. Merrell Chemical Company in 1956 and has been used for ovulation induction in infertile women with hypothalamic/pituitary anovulation and oligoovulation, including PCOS. CC contains an unequal mixture of two non-racemic isomers, zuclomifene (Z-stereoisomer, comprising ~38%) and enclomifene (E-stereoisomer, ~62%). While zuclomifene displays mild estrogenic and antigonadotropic activity and has a relatively longer half-life compared with enclomifene, enclomifene exerts rather antiestrogenic activity (particularly on the uterus as an estrogen receptor beta antagonist). Regarding ovulation induction, it remains uncertain which isomer is a more active moiety [51]. CC binds estrogen receptor alpha expressed on gonadotropin-releasing hormone neurons, which are localized in the medial preoptic area, anterior hypothalamic area, and infundibular nucleus in the hypothalamus. The binding of clomiphene to estrogen receptor alpha is more prolonged than that of estradiol. The plasma half-life of a single dose of oral CC 50 mg is 5–7 days, and the CC and its metabolites are detectable in feces as long as 6 weeks after ingestion. CC blocks the negative feedback effect of circulating endogenous estradiol and stimulates hypothalamic gonadotropin-releasing hormone pulse release, which in turn results in the activation of FSH and LH release from the anterior pituitary glands. Increased FSH levels induce follicle growth and ovulation [52].

CC resistance (anovulatory cycle up to 150 mg/day) is observed in 10–20% of infertile women with PCOS [53]. In 2003, the Netherlands group first retrospectively compared the relationship between rs6166 and 150 mg/day CC resistance between 148 normogonadotropic anovulatory infertile women (World Health Organization class II, including PCOS) aged 20 to 40 years with oligomenorrhea (menstrual cycle > 35 days) or amenorrhea (absence of menstruation for at least 6 months and onset of withdrawal bleeding after progestagen administration), serum normal FSH limits (1–10 IU/L) and 32 normo-ovulatory women aged 20 to 35 years with a regular menstrual cycle ranging from 26 to 30 days, normal body mass index between 18 and 25 kg/m^2^, without any history of endocrine disease and medication or oral contraceptives for at least 3 months before study entry. The frequency distribution was 9% for the rs6166 AA genotype (Asn680/Asn680), 40% for the AG genotype (Asn680/Ser680), and 51% for the GG genotype (Ser680/Ser680) in a group of CC-resistant women, which was similar to that of CC-ovulatory patients [54]. In the same year, another retrospective Dutch study also reported that the frequency distribution of the rs6166 AA (Asn680/Asn680), AG (Asn680/Ser680), and GG (Ser680/Ser680) genotypes was 26%, 50%, and 24%, respectively, in 193 infertile women with PCOS. The patients with the GG (Ser680/Ser680) genotype (28%) exhibited more CC resistance compared with those with the AG (Asn680/Ser680) (14%) and AA (Asn680/Asn680) (15%) genotypes (OR 0.44; 95% CI, 0.21–0.97). After adjustment for confounding factors, including age, body mass index, mean ovarian volume, hyperandrogenism, and amenorrhea, rs6166 and basal FSH levels were predictors for CC resistance [55]. According to these findings, some sub-cohorts of infertile PCOS women with the rs6166 GG (Ser680/Ser680) genotype, who are potentially at a higher risk for CC resistance, may be treated with other ovulation induction agents, such as aromatase inhibitors (Table 2).

### 4.2. rs6165/6166 and Ovarian Response/Resistance to Letrozole

Aromatase, CYP19 gene product, is a microsomal cytochrome P450 hemoprotein-containing enzyme complex superfamily that converts androstenedione to estrone and testosterone to estradiol by catalyzing three consecutive hydroxylation reactions, converting C19 androgens to aromatic C18 estrogenic steroids. Under physiological conditions, aromatase is expressed in the granulosa cells in the ovarian follicles in non-pregnant premenopausal women [64]. Letrozole (CGS 20267) is a nonsteroidal third-generation aromatase inhibitor developed in 1993 by Ciba-Geigy Limited. Letrozole inhibits the conversion of androgens to estrogens, leading to (i) increased pituitary FSH secretion by mitigating the negative estrogen feedback on the hypothalamus, and (ii) increased secondary intraovarian androgens that potentially augment follicular sensitivity to FSH [65]. Letrozole has a shorter plasma half-life (2–4 days, single oral dose of 2.5 mg) than other third-generation aromatase inhibitors, anastrozole (41–48 h, 1 mg once daily), and exemestane (27 h, 25 mg once daily), fewer anti-estrogenic effects than CC, and no detectable androgenic effects. While letrozole maintains the normal negative feedback loop by increasing concentrations of estrogens, it does not antagonize estrogen receptors in the brain, resulting in limited FSH response, small follicle atresia, and single follicular development [66]. Higher ovulation induction rates (reported as between 54.6% and 84.4%), higher pregnancy rates, and fewer adverse effects compared with CC make letrozole a preferred option in ovulation induction in anovulatory/oligo-ovulatory infertile women, particularly in those with PCOS [67].

A retrospective study demonstrated that rs6165/rs6166 affects ovarian response to letrozole in Chinese women with PCOS [56]. This study analyzed the data from 133 women with PCOS aged less than 40 years with oligomenorrhea (menstrual cycle between 35 days and 3 months) or amenorrhea (absence of menstruation for at least 3 months and hyperandrogenemia (acne or hirsutism or serum testosterone 2.5 nmol/L or more or free androgen index 5 or more), and polycystic ovaries [presence of more than 12 antral follicles (2–9 mm in diameter) or at least one ovary with a volume greater than 10 cm3, without accompanying cysts on ultrasound]. These women did not receive other pharmacologic agents for ovulation induction before undergoing ovulation induction with letrozole alone (5 mg per day) from day 2–4 of the menstrual cycle or following progestin-induced withdrawal bleeding for a total of 5 consecutive days of administration. 40 women (30.1%) were categorized as letrozole-resistant. While the rs6165 GG genotype (Thr307/Thr307) was more prevalent in the letrozole-resistant group (57.5%) compared with the letrozole-responsive group (30.11%, OR, 1.645; 95% CI 1.120–2.415; *p* = 0.003), no differences were seen in the rs6165 AA (Ala307/Ala307) and AG (Ala307/Thr307) genotypes. Moreover, the rs6166 AA genotype (Asn680/Asn680) was more common in the letrozole-resistant group (57.5%) compared with the letrozole-responsive group (34.41%, OR, 1.543; 95% CI, 1.046–2.278, *p* = 0.013), whereas any statistical difference was not seen in the rs6166 AG (Asn680/Ser680) and GG (Ser680/Ser680) genotypes. The study also confirmed a correlation between the letrozole resistance and high body mass index/longer menstrual cycle length. Adjusting these two known potential confounders for letrozole resistance, the predictive value of rs6165/6166 remained significant, reinforcing the role of rs6165/6166 analysis in predicting ovarian response to letrozole. According to these findings, some sub-cohorts of infertile women with endometriosis, such as the rs6165 GG (Thr307/Thr307) and/or rs6166 AA (Asn680/Asn680) genotypes, who are potentially at a higher risk for letrozole resistance, other agents, such as clomiphene citrate, may be prioritized for ovulation induction.

### 4.3. rs6165/6166 and Ovarian Response/Resistance to Metformin

Metformin hydrochloride (N, N-dimethyl-imido-dicarbonimidic diamide hydrochloride) is an oral biguanide antidiabetic agent, which was discovered as a by-product in the synthesis of N, N-dimethylguanidine in 1922 and introduced as a medication (Glucophage, Merck santé s.a.s, Lyon, France) in 1957. Metformin has been broadly prescribed for women with insulin-resistant PCOS, which possibly modulates FSHR activity. Metformin potentially improves insulin sensitivity, hyperandrogenism, and anovulation in these women [68].

A recent in silico study employed computational analysis to explore the structural consequences of FSHR. This study demonstrated that the rs6165/6166 affected the morphological stability of FSHR. Molecular dynamics simulations showed that metformin has a more stable confirmation with rs6165 GG (Thr307/Thr307) and AG (Ala307/Thr307) and the rs6166 GG (Ser680/Ser680) and AG (Asn680/Ser680) genotypes, suggesting that infertile PCOS women with these genotypes potentially benefit more from metformin treatment than those with the rs6165 AA (Ala307/Ala307) and rs6166 AA (Asn680/Asn680) genotypes [57]. These findings must be confirmed in clinical settings, although the re6165/rs6166 genotype-guided individualized administration of metformin holds promise for future research.

### 4.4. rs6165/6166 and Ovarian Response/Resistance to FSH Preparations in Controlled Ovarian Stimulation

FSH plays a central role in stimulating follicular growth and development, as it is manufactured as an injectable preparation and utilized for controlled ovarian stimulation (COS) in oocyte pickup cycles, as well as other infertility treatments in both females and males. The ovarian responses to exogenous FSH administration vary among individuals and/or within an individual, broadly ranging from poor to hyper-responsive. In 2011, the Bologna criteria defined poor ovarian response (POR) as (i) advanced maternal age (≥40 years) or any other risk factor for POR, (ii) a previous history of POR (≤3 oocytes in a conventional COS cycle), and (iii) an abnormal ovarian reserve test with antral follicle count (AFC) < 5–7 or serum anti-mullerian hormone (AMH) < 0.5–1.1 ng/mL [69]. The limitations of the Bologna criteria were the heterogeneity observed within the different groups classified as POR, where the subgroups were non-comparable in terms of reproductive potential [70]. The Patient-Oriented Strategies Encompassing IndividualizeD Oocyte Number (POSEIDON) stratification was developed based on a combination of quantitative and qualitative parameters, shifting from the concept of POR to that of low prognosis (defined as the cumulative live birth rate per initiated oocyte pick cycle). According to the POSEIDON stratification, four distinct low prognosis subgroups can be identified; Group 1—patients < 35 years old with adequate ovarian reserve (AFC ≥ 5 or AMH ≥ 1.2 ng/mL) and unexpected few (<4 oocytes—Subgroup 1a) or suboptimal (4–9 oocytes- Subgroup 1b) retrieved oocyte counts in COS cycles; Group 2—patients ≥ 35 years old with adequate ovarian reserve and unexpected few (Subgroup 2a) or suboptimal (Subgroup 2b) retrieved oocyte counts; Group 3—patients < 35 years old with diminished ovarian reserve (AFC < 5 or AMH < 1.2 ng/mL); Group 4—patients ≥ 35 years with diminished ovarian reserve [71]. As POSEIDON stratification indicates, age, AFC, and AMH are the essential predictors for ovarian response to gonadotropins. However, optimizing the dose of exogenous FSH, particularly the initiating dose, for ovarian response, is challenging for reproductive endocrinologists.

The association between rs6165/rs6166 and the risk of POR to gonadotropins has been inconsistent between studies [27,72,73]. A meta-analysis of 24 studies (that included six studies, 444 cases and 875 controls for rs6165, and 22 studies, 2206 cases and 3897 controls for rs6166), however, found that the rs6165 GG (Thr307/Thr307) and AG (Ala307/Thr307) genotypes increased the risk of POR compared with the AA genotype (Ala307/Ala307) (OR, 1.80, 95% CI, 1.22–2.65, *p* = 0.003), particularly in Caucasian populations in five genetic models. In addition, the rs6166 GG (Ser680/Ser680) and AG (Asn680/Ser680) genotypes increased the risk of POR compared with the AA genotype (Asn680/Asn680) (OR, 1.38, 95% CI, 1.04–1.84, *p* = 0.025), particularly in Asian populations in four genetic models [58]. These results were supported by a recent study targeting infertile POR women in the POSEIDON Groups 3 and 4, demonstrating that the combined rs6165 GG (Thr307/Thr307) and AG (Ala307/Thr307) genotypes were associated with fewer total oocyte yield, follicular output rate, and follicular oocyte index compared with the AA genotype (Ala307/Ala307). The duration of COS was shorter in the rs6165 GG (Thr307/Thr307) genotype than in the AA (Ala307/Ala307) and AG (Ala307/Thr307) genotypes, suggesting that faster follicular development in the rs6165 GG (Thr307/Thr307) genotype may represent a less favorable ovarian response. Moreover, the rs6166 GG (Ser680/Ser680) genotype was associated with higher basal FSH concentration and more doses of gonadotropin administration in COS cycles than the AA (Asn680/Asn680) genotype. The rs6166 GG (Ser680/Ser680) genotype was also associated with a lower oocyte yield, a higher cycle cancellation rate (21%), and a POR rate (36%) compared to the AA (Asn680/Asn680) and AG (Asn680/Ser680) genotypes [59].

It is noteworthy that the majority of infertile women were treated with recombinant FSH (rFSH) preparation in these studies [27,58,59,72,73]. Meanwhile, the impact of the purified/highly purified urine-derived FSH preparation (uFSH) on rs6165/rs6166 polymorphisms remains poorly understood. To explore the optimal combination of the two different FSH preparations and rs6166 in COS in oocyte pickup cycles, Lledo et al. [61,62] investigated the relationship between rs6166 and the number and maturity of the retrieved oocytes in 191 Spanish egg-donor women aged 18–35 years with good health undergoing COS in a GnRH antagonist protocol [daily subcutaneous injection of 0.25 mg cetrorelix (Cetrotide^®^, Merck-Serono, Darmstadt, Germany) starting when the leading follicle reached 14 mm in diameter) with and GnRH agonist trigger [subcutaneous injection of 0.4 mg triptorelin (Decapeptyl^®^, Ipsen Pharma, Boulogne-Billancourt, France) prior to 36 h of follicle aspiration] for two oocyte pickup cycles (382 cycles), serving as their own controls. The egg-donor women with the rs6166 GG (Ser680/Ser680) genotypes had a greater number of retrieved oocytes (18.4 vs. 16.9) and metaphase II mature oocytes (15.5 vs. 12.8) when they were stimulated with highly purified uFSH (Fostipur^®^, Angelini, Rome, Italy, starting dose of 150–300 IU/day) compared with rFSH follitropin-α (starting dose of 150–300 IU/day). On the contrary, in egg-donor women with the rs6166 AG (Asn680/Ser680) genotype, more oocytes (20.1 vs. 16.9) and metaphase II mature oocytes (17.4 vs. 14.2) were yielded with rFSH preparation. As for AA (Asn680/Asn680), no differences were found between the two FSH preparations. In addition, the recipients of oocytes from the donors with the rs6166 GG (Ser680/Ser680) and AG (Asn680/Ser680) genotypes undergoing COS with uFSH exhibited 10% higher clinical pregnancy rates compared with the donors with AG (Asn680/Ser680) genotype, although there was no statistical difference, probably due to the small sample size. This effect may be due to the longer half-life of uFSH compared with rFSH, although the biological mechanisms underlying these findings remain unclear.

To address this, Hjelmér et al. [60] conducted further in vitro experiments, in which COS-1 cells were transfected with homozygous FSHR variants and stimulated with either uFSH or rFSH; the cyclic AMP level was then measured to assess FSHR activity. COS-1 cells transfected with rs6166 GG (Ser680/Ser680) displayed higher extracellular cAMP concentration when stimulated with uFSH compared to rFSH (10 IU: 176 vs. 39 pmol/mg protein, *p* = 0.002; 90 IU: 227 vs. 58 pmol/mg protein, *p* = 0.007), whereas COS-1 cells transfected with rs6166 AA (Asn680/Asn680), no statistical difference between the effect of uFSH and rFSH. These findings indicate that the advantage of uFSH for women with the rs6166 GG (Ser680/Ser680) genotype is not only due to the longer half-life of the uFSH preparation, but also FSHR constitution and configuration. Another possible explanation is the efficacy of the potential LH activity in uFSH preparations. The results suggest that LH supplementation is most beneficial in unexpected POR women to rFSH monotherapy and in older women (36–39 years old) undergoing infertility treatment.

Hjelmér et al. [60] also enrolled 475 infertile Swedish women into a randomized controlled study. These women were aged less than 40 years with functioning bilateral ovaries/normal ovulatory cycles (26–32 days) and body mass index less than 30 kg/m^2^. Their indications for infertility treatment were tubal factor, male factor, or unexplained infertility. The exclusion criteria included current smoking, secondary infertility, serum AMH < 5 pmol/L (<0.07 ng/mL), FSH > 12 IU/L on cycle day 2–3, presence/history of endometriosis, PCOS, and POI. They were allocated to either the highly purified menopausal gonadotropin uFSH group [Menopur^®^, Ferring Pharmaceuticals, Saint Prex, Switzerland, *n* = 239, median (range) total dose 1875 IU (250–6750 IU)] or to the rFSH follitropin-α group [Gonal-F^®^, *n* = 236, median (range) total dose 1650 IU (31–4875 IU)] in their first oocyte pickup cycles. While 98% of women underwent COS with GnRH antagonist ganirelix acetate [Ganirelix^®^, Organon & Co., Jersey City, NJ, USA, or Fyremadel^®^, SUN Pharmaceutical Industries, Mumbai, Maharashtra, India] protocols in 98% of the patients, 2% underwent COS with GnRH agonist nafarelin (Synarela^®^, Pfizer Inc., New York, NY, USA) protocols. 250 micrograms of recombinant human chorionic gonadotropin (Ovitrelle^®^) were used for trigger when ≥3 follicles reached 18 mm prior to 36 h of follicle aspiration. Autologous fresh embryo transfers were performed with luteal support using 600 mg/day (200 mg, three times) micronized progesterone vaginal tablets (Lutinus^®^, Ferring Pharmaceuticals) for 14 days. Following the determination of the clinical outcomes, including the number of retrieved oocytes, cumulative clinical pregnancy rate, and live birth rate, the rs6166 genotypes were analyzed. A sub-cohort of 221 women who received optimal COS in relation to their FSHR genotypes was selected, and the pregnancy outcomes were compared with those of 991 non-genotyped women, in whom FSH preparations were chosen based on a standard clinical evaluation. They found that the sub-cohort consisting of uFSH-treated rs6166 GG (Ser680/Ser680) and AG (Asn680/Ser680) genotypes together with rFSH-treated rs6166 AA (Asn680/Asn680) genotype had a higher chance of clinical pregnancy rate (51% vs. 40%; OR: 1.40, 95% CI 1.12–1.75, *p* = 0.003) and live birth rate (40% vs. 29%; OR: 1.55, 95% CI 1.23–1.96, *p* < 0.001) than the non-genotyped women. The miscarriage rate in the genotyped women was 7% and that in the non-genotyped was 10%.

According to these findings, uFSH may be an optimal choice for ovarian stimulation in infertile women with the rs6166 GG (Ser680/Ser680) and AG (Asn680/Ser680) genotypes, whereas rFSH may be a favorable option for those with the rs6166 AA (Asn680/Asn680) genotype. More studies are awaited from the different ethnicities, races, and regions.

### 4.5. rs6165/6166 and Ovarian Response/Resistance to Adjunctive Growth Hormone Preparation for Controlled Ovarian Stimulation

In an effort to improve clinical outcomes, the addition of growth hormone has been considered as adjuvant therapy in COS in oocyte pickup cycles, particularly for infertile women with diminished ovarian reserve. Meta-analysis demonstrated that adjunctive growth hormone in COS has an uncertain effect on live birth rate and mean number of retrieved oocytes in normal ovarian responders, but it slightly increases the number of retrieved oocytes and clinical pregnancy rate in POR women, while there is an uncertain effect on live birth rate in this group [74].

Some studies reported the possibility that POR women with the rs6166 GG genotype (Ser680/Ser680) benefit more from adjunctive growth hormone treatment in COS than those with the AA (Asn680/Asn680) and AG (Asn680/Ser680) genotypes. A Romanian prospective study enrolled 125 infertile women aged 31–43 years and body mass index 18.5–30.0 with POR defined by the Bologna criteria [63]. While fifty-eight women received growth hormone in addition to the standard COS, 67 women received the standard COS only. Women with basal FSH level greater than 15 IU/L, excluding systemic lupus erythematosus, hyper/hypothyroidism, hyperprolactinemia, or having uncontrolled diabetes mellitus, and women who had been treated with androgens, LH, or antioxidant supplementation, such as coenzyme Q10 were excluded. Starting doses of rFSH were determined according to age, body mass index, AMH level, and ovarian responses in previous COS cycles. GnRH agonist long protocol [subcutaneous injection of 0.1 mg triptorelin (Decapeptyl^®^, Ipsen, Paris, France) or GnRH antagonist protocol [subcutaneous injection of 0.25 mg cetrorelix (Cetrotide^®^, Merck Serono, Darmstadt, Germany) or ganirelix (Orgalutran^®^, Organon, Jersey City, NJ, USA)] was adopted as standard COS protocols. Subcutaneous injection of 4 mg/day growth hormone (Somatropin, ^®^ Sandoz, Basel, Switzerland) was started from the second day of the IVF cycle until three dominant ovarian follicles reached a diameter greater than 17 mm, when 250 μg recombinant human chorionic gonadotropin (Ovitrelle^®^, Merck Serono) was administered to trigger oocyte maturation. Oocyte pickup was performed 34–38 h following hCG trigger. One to three fresh embryos were transferred on day three or five following oocyte retrieval with luteal phase support using 300 mg/day vaginal micronized progesterone tablets (Lutinus^®^, Ferring Pharmaceuticals, St-Prex, Switzerland), with remaining embryos being frozen. Women with the rs6166 GG genotype (Ser680/Ser680) undergoing adjunctive growth hormone treatment had higher mean mature follicles (*p* = 0.0002) and metaphase-II oocytes (*p* = 0.0005) compared with those undergoing standard COS, whereas the effect was modest among women with the AA (Asn680/Asn680) and AG (Asn680/Ser680) genotypes.

The same research group also demonstrated that infertile women undergoing recurrent implantation failure with the rs6166 GG genotype (Ser680/Ser680) benefited from adjunctive growth hormone treatment with increased fertility rate (z = −2.723, *p* = 0.0065), transferable embryos (z = −2.772, *p* = 0.0056), better-quality embryos (2.88 vs. 1.53, *p* = 0.02) and higher blastulation rate (0.50 vs. 0.33, *p* = 0.003). Further studies are awaited to elucidate if adjunctive growth hormone treatment improves the pregnancy outcomes in embryo transfer cycles [75].

### 4.6. rs6165/rs6166 and Ovarian Hyperstimulation Syndrome

Ovarian hyperstimulation syndrome (OHSS) is an iatrogenic complication that arises from the exaggerated ovarian responses in COS cycles. OHSS is characterized by cystic swelling of the ovaries and a fluid shift from the intravascular to the third space (ascites and pleural effusion) due to increased capillary permeability and ovarian neoangiogenesis, potentially leading to maternal life-threatening conditions. The onset of OHSS largely depends on the administration of human chorionic gonadotropin. The vasoactive molecules such as interleukins, tumor necrosis factor-α, endothelin-1, and vascular endothelial growth factor are involved in the development of the syndrome [76].

The 2022 Delphi Consensus stated that there is mixed evidence supporting an association between rs6166 and OHSS [77]. A retrospective study that included 586 women undergoing their first COS cycles in IVF attempt reported a positive relationship between OHSS and the rs6166 AA (Asn680/Asn680) genotype [78]. On the contrary, another retrospective study included 50 women reported that OHSS tended to be higher in the rs6166 GG genotype (Ser680/Ser680) than in the AG (Asn680/Ser680) and AA (Asn680/Asn680) genotypes, but the values did not reach a statistical difference (OR 2.67) [78]. Finally, a meta-analysis that included 16 studies and 4287 women found no evidence for an association between rs6166 and OHSS [79].

## 5. Conclusion

The limitations of the study include that (i) this is a narrative review of the expert review, and (ii) the size of the different studies cited in this review, including the meta-analyses, is relatively small. At present, rs6165/6166 analysis remains an investigational tool rather than a routine infertility practice, due to some drawbacks like most of the underlying data come from small cohorts, single-center retrospective studies, or meta-analyses with substantial heterogeneity, lack of genotype-guided large randomized trials, poor data on cost-effectiveness, and limited integration with other genetic markers such as AMH, LHR, FSH subunit-β variants.

The genotype-guided choice of individualized ovulation stimulation preparations and protocols, however, has great potential to reduce unexpected poor or high ovarian responses in ovulation induction/ovarian stimulation and improve clinical outcomes in reproductive medicine, as recent breakthroughs in comprehensive genomic profiling contribute to the choice of anticancer agents and the assessment of their safety in cancer treatment. As the allele frequency of rs6165/6166 exhibits racial and ethnic variances, clinical studies from different populations are anticipated for the realization of the individualized ovulation induction, ovarian stimulation, and precision medicine in infertility treatment. In addition, multi-locus pharmacogenetic scores for gonadotropin dosing, prospective studies on rs6165/6166-guided choice between rFSH and uFSH preparations, and validation of metformin–FSHR interactions in PCOS cohort are intriguing fields of research. Further studies are warranted to ascertain the potential of rs6165/6166 analysis for clinical implementation.

## Figures and Tables

**Table 2:** Association between FSHR polymorphisms rs6165/6166 and ovarian response/resistance to oral ovulation induction agents, FSH preparations, and adjunctive growth hormone.

	rs6165	rs6166
Clomiphene citrate	Not investigated	GG (Ser680/Ser680); potentially associated with ovarian resistance (case–control studies) [54,55]
Letrozole	GG (Thr307/Thr307); potentially associated with ovarian resistance (case–control studies) [56]	AA (Asn680/Asn680); potentially associated with ovarian resistance (case–control studies) [56]
Metformin	GG (Thr307/Thr307) and AG (Ala307/Thr307); potentially associated with improved ovarian response? [57]	GG (Ser680/Ser680) and AG (Asn680/Ser680); potentially associated with improved ovarian response? [57]
Recombinant FSH	GG (Thr307/Thr307) and AG (Ala307/Thr307); potentially associated with ovarian resistance, particularly in Caucasian populations (meta-analysis) [58,59]	GG (Ser680/Ser680) and AG (Asn680/Ser680); potentially associated with ovarian resistance, particularly in Asian populations (meta-analysis) [58,59] AA (Asn680/Asn680); potentially associated with improved clinical pregnancy rate and live birth rate (randomized controlled study) [60]
Urine-derived FSH	Not investigated	GG (Ser680/Ser680) and AG (Asn680/Ser680); potentially associated with improved clinical pregnancy rate and live birth rate (case–control studies and randomized controlled study) [60,61,62]
Adjunctive growth hormone	Not investigated	GG genotype (Ser680/Ser680); potentially associated with improved ovarian response [63]

## Data Availability

No new data were created or analyzed in this study .
